# Assessment of verbal and visuospatial working memory in mild cognitive impairment and Alzheimer's dementia

**DOI:** 10.1590/1980-57642015DN93000014

**Published:** 2015

**Authors:** Roy P.C. Kessels, Anouk Overbeek, Zita Bouman

**Affiliations:** 1 Professor of neuropsychology (PhD) at Donders Institute for Brain, Cognition and Behaviour, Radboud University, Nijmegen, the Netherlands; Clinical Neuropsychologist at the Department of Medical Psychology, Radboud University Medical Center, Nijmegen; Scientific advisor at Vincent van Gogh Institute for Psychiatry, Venray, the Netherlands; Visiting professorial fellow at the School of Psychology, University of New South Wales, Sydney, Australia.; 2 Psychologist-intern (MSc) at the Department of Medical Psychology, Radboud University Medical Center, Nijmegen, the Netherlands and junior researcher at the Department of Public Health, Erasmus MC, Rotterdam, the Netherlands.; 3 Psychologist (MSc) and junior researcher at Donders Institute for Brain, Cognition and Behaviour, Radboud University, Nijmegen, the Netherlands and Kempenhaeghe, Academic Centre for Epileptology, Heeze, the Netherlands

**Keywords:** working memory, Wechsler memory scale, spatial addition, spatial span, digit span

## Abstract

In addition to episodic memory impairment, working memory may also be compromised in mild cognitive impairment (MCI) or Alzheimer's dementia (AD), but standard verbal and visuospatial span tasks do not always detect impairments.

**Objective:**

To examine whether more complex verbal and visuospatial working memory tasks result in more reliable impairment detection.

**Methods:**

The Digit Span (forward, backward and sequencing), Spatial Span (forward and backward) and Spatial Addition test from the Wechsler batteries were administered to MCI and AD patients and performance compared to healthy older adult controls.

**Results:**

Results showed that both the MCI and AD patients had impaired performance on the Spatial Addition test. Both groups also had impaired performance on all three Digit Span conditions, but no differences were found between forward and backward conditions in any of the groups. The sequencing condition differed from the backward condition only in the AD group. Spatial Span performance was impaired in AD group patients but not in MCI patients.

**Conclusion:**

Working memory deficits are evident in MCI and AD even on standard neuropsychological tests. However, available tests may not detect subtle impairments, especially in MCI. Novel paradigms tapping the episodic buffer component of working memory may be useful in the assessment of working memory deficits, but such instruments are not yet available for clinical assessment.

## Abstract (pt)

Além do comprometimento da memória episódica, a memória de trabalho (ou memória operacional) também pode ser afetada no comprometimento cognitivo leve (CCL) ou na demência de Alzheimer (DA), mas as tarefas padrão de extensão verbal e de extensão visuoespacial nem sempre detectam deficiências.

**Objetivo:**

Investigar se tarefas mais complexas de memória de trabalho verbais e visuoespaciais são mais confiáveis para detectar comprometimento.

**Métodos:**

Os testes de extensão de dígitos (em ordem direta, inversa e sequencial), extensão espacial (ordem direta e inversa), e o teste de adição espacial das baterias Wechsler foram administradas a pacientes com CCL e DA e o desempenho foi comparado aos de controles idosos saudáveis.

**Resultados:**

Os resultados mostraram que tanto pacientes com CCL ou DA tiveram prejuízo no desempenho no teste adição espacial. Tanto os pacientes com CCL como os de DA também tiveram desempenho prejudicado em todos os três testes de extensão de dígitos, mas não foram encontradas diferenças entre a ordem direta e inversa em qualquer dos grupos. A condição sequencial diferia da condição inversa apenas no grupo DA. O desempenho no teste de extensão espacial foi comprometido em pacientes do grupo DA, mas não em pacientes com CCL.

**Conclusão:**

Os déficits de memória de trabalho são evidentes no CCL e DA, mesmo em testes neuropsicológicos padrão. No entanto, os testes disponíveis podem não detectar deficiências sutis, especialmente no CCL. Novos paradigmas que utilizem o componente retentor episódico da memória de trabalho pode ser útil na avaliação dos déficits de memória de trabalho, mas esses instrumentos ainda não estão disponíveis para avaliação clínica.

## INTRODUCTION

Alzheimer's disease patients typically present with profound long-term memory deficits. These impairments are already evident in the pre-dementia stage of the disease, referred to as Mild Cognitive Impairment,^1^ and become more profound in the Alzheimer dementia (AD) stage, in which everyday functioning is also affected. Consequently, long-term memory function has been extensively studied in MCI and AD, yet less attention has been paid to working memory.

In Baddeley's widely used model, working memory is the ability to maintain and manipulate information for a brief period of time. The model consists of two slave systems: the phonological loop for verbal information and the visuospatial sketchpad for visual and spatial information.^2^ The capacity of the phonological loop is typically assessed using digit span tasks, while the visuospatial sketchpad can be assessed using spatial span tests.^3^ Both working memory components function under the control of the Central Executive (CE) which is recruited under higher memory loads. For example, backward span taps the CE to a greater extent than forward span.

Results for deficits in the phonological loop in MCI are mixed. Several studies have reported unimpaired performance on digit span forward and backward tasks,^4-6^ whereas others have shown deficits on both the digit span forward and backward in MCI patients.^7^ Spatial span performance has been found not to differ between MCI patients and healthy controls, for neither forward nor backward span.^8,9^ In AD patients, most studies show deficits in digit span performance compared to healthy older adults.^9-11^ Only a few studies have examined spatial span performance in AD, showing that AD patients have a lower forward and backward spatial span performance than both older adult controls and MCI patients.^8,9,11^

One reason for not finding impairments on digit or spatial span tasks in patients with less severe deficits (i.e., in MCI) may be that the tasks at hand are not sufficiently difficult, resulting in an unimpaired performance compared to healthy controls even for backward span tests. While working memory complexity has often been manipulated using experimental paradigms, these tasks lack standardisation and normative data for use in clinical settings. The aim of the present study was therefore to examine working memory performance in MCI and AD using more complex, yet clinically available, working-memory tasks from the Wechsler batteries, in addition to the regular forward and backward span tasks.

## METHODS

A total of 50 individuals participated in this study. Twenty-five patients from the memory clinic of Radboud University Medical Center, Nijmegen participated. Patients fulfilled the established clinical criteria for MCI^1^ (N=11) or AD^12^ (N=14). Twenty-five healthy older adults without a history of neurological or psychiatric disease and no subjective cognitive complaints were recruited as control participants. Table 1 shows the participants' characteristics. The groups did not differ with respect to age (F(2,47)=1.0), sex distribution (Χ^2^(2)=1.1) or education level (Χ^2^(2)=4.4). Written informed consent was obtained from all participants in accordance with the declaration of Helsinki. The study was exempt from formal medical-ethical approval, since the tasks are brief, clinically available and administered as part of routine neuropsychological assessment, thus not burdening the patients or their caregivers.

**Table 1:** Characteristics (mean+SD or frequencies) of the MCI, AD and control group participants.

	MCI	AD	Controls
Age	78.3 (5.8)	77.1 (7.7)	74.8 (7.9)
Sex (m:f)	6:5	6:8	9:16
Educational level (low:middle:high)	2:3:6	3:8:3	1:11:13
MMSE	26.7 (1.0)	19.00 (3.3)	29.4 (0.8)

MCI: Mild Cognitive Impairment; AD: Alzheimer’s dementia; MMSE: Mini-Mental State Examination.

Three working memory tests were administered. In the patient group, the results of these working memory tests were not used in the diagnostic process. First, the Digit Span subtest from the Wechsler Adult Intelligence Scale – Fourth Edition (WAIS-IV)^13^ was administered to assess verbal working memory. This task consists of three conditions. In the forward condition, sequences of digits of increasing length have to be repeated in the same order as presented. In the backward condition, digit sequences have to be repeated in reverse order. In the most complex sequencing condition, sequences of digits of increasing length have to be sorted into numerical order.

For assessing visuospatial working memory, the Spatial Span subtest from the Wechsler Memory Scale – Third Edition (WMS-III)^14^ was administered. This subtest is similar to the Corsi Block-Tapping Task and consists of a board with ten spatially distributed cubes mounted on top of it. The examiner taps block sequences of increasing length that have to be repeated in the same (forward) or reverse (backward) order. As a more complex measure of visuospatial memory, the subtest Spatial Addition from the WMS-IV^15^ was administered. This test is based on the principle of the n-back paradigm. In this task, a grid is briefly presented, which contains blue and/or red circles located in some of the cells of the grid. Subsequently, a second grid with blue and/or red circles is shown. The participant must then add or subtract both displays using a set of rules. If two blue circles appear in the same cell subsequently, they have to be subtracted, and the participant must place a white circle in that cell in an (empty) response grid. If a blue circle appears only once in a particular location, the participant must place a blue circle in that cell. Additionally, any red circles have to be ignored. The difficulty level of the items gradually increases.

## RESULTS

Table 2 shows the results on the working memory tests. Violations of the normality assumption were checked using Shapiro-Wilk's test. As a result, one variable (Digit Span backward) was transformed by square-root transformation, resulting in a normally distributed variable that was used in subsequent analyses unless stated otherwise. Multivariate General Linear Model analyses of variance resulted in group differences on all working memory variables (all F-values>3.7, p-values <0.032). Dunnett post-hoc comparisons showed that the MCI group performed worse than the healthy controls on Digit Span forward, backward and sequencing (p=0.029, p=0.02, p=0.026, respectively) as well as on Spatial Addition (p<0.0005), but not on Spatial Span. AD patients performed worse than controls on all working memory variables (all p-values <0.014). Effect sizes were all in the large range for the AD group and in the medium-to-large range for the MCI group. Comparing forward and backward performance, backward Spatial Span was lower than forward span only in the AD group (t(13)=2.9, p=0.012). No differences between forward and (untransformed) backward Digit Span were found in any of the groups (all t-values<1.9). Digit Span sequencing was associated with worse performance than Digit Span backward (untransformed) in the AD (t(13)=2.7, p=0.018), but not in any of the other groups (t-values<1.0). Post-hoc comparisons between the MCI and AD patients revealed a significantly worse performance in the AD group for Spatial Addition (p=0.014), Spatial Span backward (p=0.008), as well as Digit Span backward (p=0.028) and sequencing (p=0.002).

**Table 2:** Performance (mean, SD and Cohen’s d) on the Spatial Span, Digit Span and Spatial Addition subtests for the MCI, AD and control group participants.

		MCI				AD				Controls	
		Mean	SD	D		Mean	SD	D		Mean	SD
Spatial Span (WMS-III)	Forward	5.6	1.3	–0.69		5.2*	2.1	–0.81		6.8	1.9
	Backward	6.1	1.6	–0.24		3.9**	1.9	–1.29		6.6	2.2
Digit Span (WAIS-IV)	Forward	7.5*	1.6	–0.78		6.4**	1.5	–1.52		8.7	1.5
	Backward	7.0*	1.2	–0.85		5.6**	1.6	–1.55		8.6	2.1
	Sequencing	6.4*	1.6	–0.94		3.6**	2.2	–2.12		8.4	2.3
Spatial Addition (WMS-IV)		7.4*	4.0	–0.96		4.8**	2.1	–1.80		9.7	3.0

MCI: Mild Cognitive Impairment; AD: Alzheimer’s dementia; WMS: Wechsler Memory Scale; WAIS: Wechsler Adult Intelligence Scale;

* p<0.05;

** p<0.001 (control group as reference).

## DISCUSSION

The present study clearly showed that working memory deficits are present in both MCI and AD, but that impairments are not always evident on all working memory tests, i.e. MCI patients do not show deficits on the Spatial Span, in agreement with previous results,^8^ no difference between forward and backward performance was found on this test in the MCI and control groups. One explanation for this may be that the same sequences are used for forward and backward testing, thus promoting incidental or implicit learning.^16^ However, a similar finding was present in healthy participants on the Corsi, which uses different sequences for forward and backward testing.^3^ Probably, the working memory load of the Spatial Span test is limited, making it less susceptible to subtle impairments. A visuospatial working memory test which showed reliable deficits is the Spatial Addition subtest, on which deficits were found in both the MCI and AD group. Since participants have to maintain and manipulate information from previous presentations (as in the n-back paradigm), the working memory load is higher, requiring greater CE recruitment.

With respect to the Digit Span, MCI and AD patients performed worse on all conditions. Significant differences between backward and sequencing performance, but not between forward and backward performance were found only in the AD group, whereas MCI and healthy participants showed no evidence of increased working memory load on the Digit Span. These results are in line with recent findings demonstrating that the differences in level of processing between Digit Span forward, backward and sequencing were too small to separate complex, executive processing from passive, slave-system storage.^17^

From a clinical perspective, working memory assessment is important. For instance, working memory performance has predictive value in MCI patients with respect to conversion to dementia.^18-19^ Although the present findings showed that deficits are present in verbal and visuospatial memory, not all currently available working memory tests reveal impairments. A promising approach may lie in novel working memory paradigms that rely on the episodic buffer. In Baddeley's updated working memory model,^20^ the episodic buffer was introduced, which integrates information from different modalities, can act as an overflow buffer if information exceeds the capacity of the slave systems, and interacts with long-term working memory. Indeed, impairments on experimental working-memory paradigms that require the integration of object with location or shape information have been found in MCI,^21,22^ AD^23^ and non-symptomatic Alzheimer's disease.^24^ Thus, episodic buffer tasks in the assessment of early Alzheimer patients are highly relevant.^9^ One could argue that on the Spatial Addition test, objects (coloured circles) have to be combined with their location and maintained over time, possibly tapping the binding aspect of the episodic buffer. Moreover, the high-load trials of this test may exceed the capacity of the visuospatial sketchpad, recruiting the episodic buffer's overflow function. However, the exact contribution of slave system, CE or episodic buffer processing is difficult to disentangle using the currently available standardized tests.^25^ This shortcoming illustrates the need for new episodic buffer tasks that specify in detail which theoretical working memory construct is being assessed,^25^ with good psychometric properties for use in clinical assessment.
